# Attitude Controller for UAV First-Order Acceleration Coefficients Tracking

**DOI:** 10.3390/s26092727

**Published:** 2026-04-28

**Authors:** Ding Xu, Hailong Pei

**Affiliations:** 1 Key Laboratory of Autonomous Systems and Network Control, Ministry of Education, South China University of Technology, Guangzhou 510641, China; auxuding1997@mail.scut.edu.cn; 2 Guangdong Engineering Technology Research Center of Unmanned Aerial Vehicle Systems, South China University of Technology, Guangzhou 510641, China; 3 School of Automation Science and Engineering, South China University of Technology, Guangzhou 510641, China

**Keywords:** UAV, attitude controller, quaternion decomposition

## Abstract

This paper proposes a novel attitude control methodology for unmanned aerial vehicles (UAVs). The core of the approach is a decomposition of the vehicle’s orientation into a distinct horizontal tilt component and a yaw rotation. This reformulation provides a principled theoretical framework by redefining attitude error within an acceleration-coefficient space, which linearizes and decouples the horizontal acceleration dynamics—a significant simplification over conventional Euler-angle or quaternion-based models. From an engineering perspective, this framework enables the direct generation of angular velocity commands that guarantee first-order tracking of desired acceleration coefficients. This leads to a substantial reduction in the computational complexity of the outer-loop controller, enhances trajectory tracking accuracy, and ensures predictable performance under given attitude constraints and angular velocity limits. Simulation results demonstrate the method’s superior performance compared to standard techniques, validating its dual value as both a theoretically-grounded control design tool and an effective solution for high-precision UAV navigation in real-world applications.

## 1. Introduction

Common non-fixed-wing Vertical Take-Off and Landing (VTOL) aircraft, such as multi-rotor systems and helicopters, control their position and orientation in three-dimensional space by adjusting attitude and thrust magnitude. An attitude controller computes the inputs required to drive the vehicle from its current orientation toward a desired attitude. The method of attitude representation and the definition of attitude error jointly determine the trajectory from the present to the target orientation.

In many contemporary attitude control designs, the error for each channel is typically derived from the difference between a single attitude parameter and its desired value. When Euler angles are used for attitude representation, control strategies often account for rigid-body rotation dynamics to reduce coupling among parameters, thus treating attitude control as a set of single-channel problems [1,2,3]. A variety of control methods have been applied to Euler-angle-based attitude representations, such as ADRC, fuzzy control, and sliding mode control [4,5,6,7,8,9,10]. However, such Euler-angle-based approaches can encounter singularities and increased nonlinear coupling during large-angle maneuvers, as noted in surveys on attitude control strategies [11].

The kinematic model of a UAV is fundamentally built upon the overall relationships among angular velocity, attitude, acceleration, velocity, and position. Therefore, treating the attitude-dependent coefficients in the acceleration terms as control objectives and decoupling the interactions between different acceleration channels is a more advantageous approach [12,13]. This method not only simplifies and partially linearizes the outer-loop mathematical model but also generates attitude trajectories with clear physical meaning [14]. In recent years, control methods based on SO(3) or quaternions (such as geometric control) have achieved a certain degree of decoupling and theoretically guaranteed global stability [8,15,16,17,18,19]. Compared to SE(3), NMPC for outer-loop control can better handle the physical constraints of states and control inputs. However, the high computational cost of optimization limits its application in embedded deployment to only the outer loop, where the inner-loop dynamics are estimated to generate attitude sequences for inner-loop control [20,21].

To design a more suitable inner-loop controller for the outer-loop NMPC control, and through the design goal of a first-order approximation of the acceleration coefficient, the inner-loop model in the outer-loop NMPC is made more accurate while saving parameters and optimization computational effort. This study derives a novel attitude error calculation method and generates corresponding trajectories. Given that the yaw angle is typically treated as an independent control objective decoupled from position control, we propose a new computational framework using quaternions that separates the yaw parameters from the roll and pitch parameters. During attitude calculation, roll and pitch are considered together to decouple the interactions between multiple acceleration channels. The designed attitude trajectory is suitable for attitude ranges with tilt angles less than π/2, and can achieve first-order tracking of the horizontal dual-channel acceleration coefficients under given attitude constraints and angular velocity limits, thereby providing a more concise mathematical representation for the outer-loop model. This approach is based on and extends previous research work on nonlinear decoupling and quaternions.

## 2. Mathematical Model of UAV

Common underactuated mathematical models for non-fixed-wing vertical take-off and landing (VTOL) aircraft share some notable structural similarities, including quadrotors, helicopters, and ducted fan drones. The core characteristic of an underactuated system is that the number of control inputs is fewer than the system’s degrees of freedom, typically featuring 6 degrees of freedom (3 translational + 3 rotational) but only 4 control inputs. The control of these UAVs often adopts a hierarchical design, divided into attitude control (inner loop) and position control (outer loop) [22]. The desired attitude angles and total thrust serve as virtual inputs for the outer loop.

And these VTOL aircraft mathematical models all share similar kinematic and dynamic frameworks, with the main distinction lying in how torque and lift are generated. Here, we abstract the control inputs as torque and total thrust. Taking the equilibrium point as the origin of the body attitude, the total thrust is oriented vertically upward in the body-fixed coordinate system.

### 2.1. Dynamics and Kinematics

Let the world coordinate frame $F_{w}$ and the body coordinate frame $F_{b}$ be defined as right-handed systems. In the world coordinate system, the translational dynamics equations of the VTOL aircraft can be expressed as follows:

(1) $$m\dot{V}=m{\left[0,0,g\right]}^{T}+R{\left[0,0,-T\right]}^{T}+F_{drag,disv}$$

where *m* denotes the mass of the UAV, V represents the velocity in the North-East-Down (NED) coordinate frame, R is the rotation matrix of the vehicle attitude, *T* is the total thrust from the rotors, and $F_{drag,disv}$ is the aerodynamic drag and other disturbances.

In the body-fixed coordinate system, the rotational dynamics equation of the VTOL aircraft can be expressed as:

(2) $$J\dot{\omega }=\tau -\omega \times \left(J\omega \right)$$

where J denotes the inertia matrix of the vehicle, τ represents the three-axis torque, and ω is the angular velocity.

In the world coordinate system, the position kinematics equation of the VTOL aircraft can be expressed as follows:

(3) $$\dot{P}={\left[\dot{N},\dot{E},\dot{D}\right]}^{T}=V={\left[u,v,w\right]}^{T}$$

Construct the mathematical model of the UAV in the sequence of position-velocity-acceleration-attitude-angular velocity. Considering the conventional flight attitudes of this type of aircraft, this paper excludes inverted flight conditions.

### 2.2. Attitude Representation and Acceleration Coefficients

Two commonly used attitude representations for UAVs are Euler angles and quaternions. They primarily influence the horizontal acceleration coefficients through the rotation matrix R defined in Equation (1).

Using the commonly adopted ‘Z-Y-X’ rotation sequence of Euler angles ${\left[\phi ,\theta ,\psi \right]}^{T}$ to represent the vehicle attitude, R(ϕ,θ,ψ) represent the rotation matrix. The rotational kinematics of the VTOL aircraft can be expressed using Euler angles as follows:

(4) $$\dot{V}=\left[\begin{array}{c} \dot{u}\\ \dot{v}\\ \dot{w} \end{array}\right]=\left[\begin{array}{c} T/m\cdot (\cos\varphi \sin\theta \cos\psi +\sin\varphi \sin\psi )\\ T/m\cdot (\cos\varphi \sin\theta \sin\psi -\sin\varphi \cos\psi )\\ g-T/m\cdot (\cos\varphi \cos\theta ) \end{array}\right]$$

The attitude kinematics equation of the VTOL aircraft can be expressed using Euler angles as follows:

(5) $$\left[\begin{array}{c} \dot{\phi }\\ \dot{\theta }\\ \dot{\psi } \end{array}\right]=\left[\begin{array}{ccc} 1 & 0 & -\sin\theta \\ 0 & \cos\phi & \sin\phi \cos\theta \\ 0 & -\sin\phi & \cos\phi \cos\theta \end{array}\right]\left[\begin{array}{c} p\\ q\\ r \end{array}\right]$$

Using quaternions $q={\left[w_{0},x_{0},y_{0},z_{0}\right]}^{T}$ to represent attitude, R(q) represent the rotation matrix. The rotational kinematics of the VTOL aircraft can be expressed using quaternions as follows:

(6) $$\dot{V}=\left[\begin{array}{c} \dot{u}\\ \dot{v}\\ \dot{w} \end{array}\right]=\left[\begin{array}{c} T/m\cdot (2w_{0}y_{0}+2x_{0}z_{0})\\ T/m\cdot (2y_{0}z_{0}-2w_{0}x_{0})\\ g-T/m\cdot (1-2x_{0}^{2}-2y_{0}^{2}) \end{array}\right]$$

The attitude kinematics equation of the VTOL aircraft can be expressed using quaternions as follows:

(7) $$\dot{q}=\frac{1}{2}q⊗\omega$$

The Euler angle representation suffers not only from the gimbal lock problem but also from induced heading changes during purely oblique tilting maneuvers. The quaternion representation, on the other hand, is constrained by its four interdependent parameters (subject to the unity-norm condition), which are not fully independent. Furthermore, in both methods, the horizontal acceleration coefficients are mutually coupled and nonlinear. When combined with the rigid-body rotational dynamics, this makes it difficult to directly map the desired acceleration coefficients to angular velocity commands for achieving first-order tracking of the acceleration coefficients [12].

### 2.3. Attitude Decomposition

The subsequent analysis utilizes two specific decompositions of the vehicle’s attitude, each constituting a horizontal tilt ${\left[a,x,y,0\right]}^{T}$ and a rotation about the z-axis ${\left[b,0,0,z\right]}^{T}$, with a>0 and b>0. These decompositions differ in their order of operations: one is a tilt-yaw sequence, and the other is a yaw-tilt sequence. With inverted flight excluded, we have $a>\sqrt{1/2}$.

The vehicle attitude representation is ${\left[w_{0},x_{0},y_{0},z_{0}\right]}^{T}$. The attitude representation under the tilt-yaw decomposition is given by:

(8) $${\left[\begin{array}{cccc} a & x & y & 0 \end{array}\right]}^{T}⊗{\left[\begin{array}{cccc} b & 0 & 0 & z \end{array}\right]}^{T}={\left[\begin{array}{cccc} w_{0} & x_{0} & y_{0} & z_{0} \end{array}\right]}^{T}$$

Solving for the decomposed parameters yields:

(9) $$\left[\begin{array}{c} a\\ b\\ x\\ y\\ z \end{array}\right]=\left[\begin{array}{c} \sqrt{w_{0}^{2}+z_{0}^{2}}\\ w_{0}/a\\ (w_{0}x_{0}-z_{0}y_{0})/a\\ (z_{0}x_{0}+w_{0}y_{0})/a\\ z_{0}/a \end{array}\right]$$

Substituting into Equation (6), we obtain:

(10) $$\dot{V}=\left[\begin{array}{c} a_{N}\\ a_{E}\\ a_{D} \end{array}\right]=\left[\begin{array}{c} T/m\cdot 2ay\\ T/m\cdot (-2ax)\\ g-T/m\cdot (1-2x^{2}-2y^{2}) \end{array}\right]$$

Analysis of Equation (10) shows a direct correlation between the a,x,y term in the tilt-yaw decomposition and the translational dynamics. This correlation has a clear physical basis: the UAV’s acceleration is governed exclusively by the tilt of the thrust vector, while rotation about the z-axis (yaw) is dynamically decoupled from translational acceleration.

**Figure 1:**
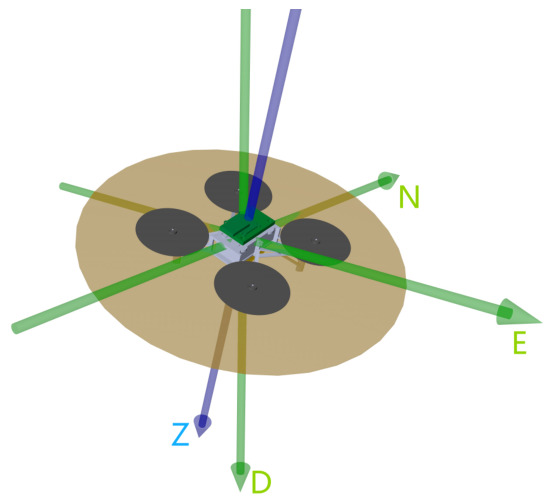
The influence of VTOL attitude on horizontal acceleration in the North-East-Down (NED) coordinate frame.

As shown in Figure 1, the horizontal acceleration along the north and east directions is only related to the thrust and the tilt angle of the z-axis in body frame, and is independent of the heading. In the North-East-Down (NED) coordinate frame, the result of rotating the D-axis by the quaternion ${\left[a,x,y,0\right]}^{T}$ yields the body z-axis.

Consequently, by taking ax and ay as the control objectives for attitude, the downward acceleration is $a_{D}=g-T/m\cdot \sqrt{1-4{\left(ax\right)}^{2}-4{\left(ay\right)}^{2}}$. Moreover, the attitude controller utilizes the error signal derived from the difference between the desired value $ax_{d}$ and its measured value ax. An identical error computation method is applied to the lateral acceleration ay.

**Proof.**  Substitution of the quaternion properties into a,x,y gives:

$$\begin{array}{cc} a^{2}+x^{2}+y^{2} & =1 \end{array}$$

Multiply both sides by $a^{2}$ and transpose the terms to the left-hand side.

$$\begin{array}{cc} a^{4}-a^{2}+\left[{\left(ax\right)}^{2}+{\left(ay\right)}^{2}\right] & =0 \end{array}$$

With a>0, Solving the equations yields:

(11) $$a=\sqrt{\frac{1+\sqrt{1-4{\left(ax\right)}^{2}-4{\left(ay\right)}^{2}}}{2}}$$

Substituting the quaternion properties into Equation (10), followed by insertion into the above equation, gives:

(12) $$\begin{array}{cc} a_{D} & =g-T/m\cdot (2a^{2}-1)\\ \; & =g-T/m\cdot \sqrt{1-4{\left(ax\right)}^{2}-4{\left(ay\right)}^{2}} \end{array}$$

   □

The attitude representation under the yaw-tilt decomposition is given by:

(13) $$\left[\begin{array}{c} b\\ 0\\ 0\\ z \end{array}\right]⊗\left[\begin{array}{c} a\\ x\\ y\\ 0 \end{array}\right]=\left[\begin{array}{c} ab\\ bx-yz\\ by+xz\\ az \end{array}\right]=\left[\begin{array}{c} w_{0}\\ x_{0}\\ y_{0}\\ z_{0} \end{array}\right]$$

With the quaternion properties $b^{2}+z^{2}=1$:

(14) $$\begin{array}{c} a^{2}b^{2}+a^{2}z^{2}=a^{2}=w_{0}^{2}+z_{0}^{2} \end{array}$$

With a>0, $a=\sqrt{w_{0}^{2}+z_{0}^{2}}$. Solving for the decomposed parameters yields:

(15) $$\left[\begin{array}{c} a\\ b\\ x\\ y\\ z \end{array}\right]=\left[\begin{array}{c} \sqrt{w_{0}^{2}+z_{0}^{2}}\\ w_{0}/a\\ (w_{0}x_{0}+z_{0}y_{0})/a\\ (-z_{0}x_{0}+w_{0}y_{0})/a\\ z_{0}/a \end{array}\right]$$

The yaw-tilt decomposition is akin to the standard yaw-pitch-roll Euler angle formulation. This analogy is grounded in the underlying physics: owing to their strong dynamic coupling and identical mechanism for moment generation, roll and pitch are aggregated into a single horizontal tilt component, denoted ${\left[a,x,y,0\right]}^{T}$. Whereas, the yaw moment, generated by a fundamentally different mechanism, is represented by a separate term ${\left[b,0,0,z\right]}^{T}$.

The mathematical representation of the yaw component is common to both decompositions.

## 3. Attitude Planning for Tracking First-Order Acceleration

In common inner-loop attitude controllers for UAVs, the attitude error between the current and desired orientation is computed based on the difference of a single parameter from the chosen attitude representation (typically Euler angles or quaternions). This derived error is then fed into the attitude dynamics equations to obtain the desired angular velocity.

### 3.1. Design Objective

A fundamental challenge arises when simplified attitude control couples with translational dynamics. If we model the attitude loop as an ideal first-order system (ignoring angular rate dynamics and delays), the resulting horizontal acceleration becomes a nonlinear function of the attitude command. A clear example is rolling from [roll, pitch, yaw] to [-roll, pitch, yaw]: this not only reverses the lateral acceleration but also introduces an undesired longitudinal component. This nonlinearity motivates the design of a new method for generating desired angular velocity that directly facilitates first-order tracking of the desired acceleration vector.

The outer-loop Nonlinear Model Predictive Control (NMPC) generates a desired attitude command sequence at a low frequency based on position, velocity, and attitude. The inner loop consists of an angular velocity closed-loop controller. This paper focuses on designing an attitude-to-angular-velocity controller that converts the desired attitude into a desired angular velocity, using quaternions as the attitude representation.

Due to the limited computational frequency of the NMPC algorithm on the onboard processor, we aim for the attitude trajectory to track changes in acceleration linearly. This approach is intended to reduce the computational burden on the NMPC and improve overall control performance.

### 3.2. Acceleration Decoupling Compensation

In the tilt-yaw decomposition, assuming the angular velocity corresponding to ${\left[a,x,y,0\right]}^{T}$ during the attitude change is ${\left[0,R_{x},R_{y},R_{z}\right]}^{T}$.

In Equation (10), the three-axis accelerations of the UAV are determined by [ax,ay,T]. From the perspective of control allocation, [ax,ay,T] are primarily used to control $\left[a_{N},a_{E},a_{D}\right]$, respectively. Due to the moment of inertia being significantly smaller than the mass and the performance of gyroscopes being substantially higher than that of accelerometers, the UAV’s moment control loop is generally much faster than that of the total thrust control loop. And since the value of *T* typically fluctuates around the gravitational acceleration g, *T* can be approximated as a constant in $a_{N}$ and $a_{E}$ over short time intervals.

It is known that $a_{N}$ (North acceleration) depends only on ay, and $a_{E}$ (East acceleration) depends only on ax. However, it is evident that due to the nature of the quaternion derivative, $\dot{a_{N}}$ and $\dot{a_{E}}$ are not decoupled. In practice, a PD controller may also be employed; however, the following discussion uses a simple P controller for illustrative purposes. For example, if $a_{N}$ follows a first-order trajectory $\dot{a_{N}}=p\left(a_{Nd}-a_{N}\right)$, then the required $R_{y}^{N}=Kp\left[{\left(ay\right)}_{d}-ay\right]$, where ${\left(ay\right)}_{d}$ represents the desired value of ay. And it is necessary to enforce $\dot{a_{E}}=0$.

With the quaternion properties

(16) $$\left[\begin{array}{c} \dot{a}\\ \dot{x}\\ \dot{y}\\ \dot{z} \end{array}\right]=\left[\begin{array}{c} (-x\omega_{x}-y\omega_{y}-z\omega_{z})/2\\ (a\omega_{x}-z\omega_{y}+y\omega_{z})/2\\ (z\omega_{x}+a\omega_{y}-x\omega_{z})/2\\ (-y\omega_{x}+x\omega_{y}+a\omega_{z})/2 \end{array}\right],\left[\begin{array}{c} \omega_{x}\\ \omega_{y}\\ \omega_{z} \end{array}\right]=\left[\begin{array}{c} R_{x}^{N}\\ R_{y}^{N}\\ R_{z}^{N} \end{array}\right]$$

(17) $$\begin{array}{cc} \dot{a_{E}} & ={\left(-2wx+2yz\right)}^{\prime }\\ \; & =2a\dot{y}+2y\dot{a}+2x\dot{z}+2z\dot{x}\\ \; & =R_{x}^{N}\left(-a^{2}+x^{2}-y^{2}+z^{2}\right)+2R_{y}^{N}\left(az+xy\right)=0 \end{array}$$

Solving the equations yields the decoupled compensation amount:

(18) $$R_{x}^{N}=\frac{2(az+xy)}{a^{2}-x^{2}+y^{2}-z^{2}}R_{y}^{N}$$

Similarly, when $a_{N}$ follows the first-order trajectory $\dot{a_{E}}=P\left(a_{Ed}-a_{E}\right)$, we obtain:

(19) $$R_{y}^{E}=\frac{2(xy-az)}{a^{2}+x^{2}-y^{2}-z^{2}}R_{x}^{E}$$

Furthermore, rotations $R_{x}$ and $R_{y}$ alter the initial yaw component of ${\left[a,x,y,0\right]}^{T}$. Imposing the constraint that the initial yaw within ${\left[a,x,y,0\right]}^{T}$ must remain invariant:

(20) $${\dot{z}}_{axy}=\frac{1}{2}\left(aR_{z}+xR_{y}-yR_{x}\right)=0$$

Thus, ${\left[R_{x},R_{y},R_{z}\right]}^{T}$ becomes:

(21) $$\left[\begin{array}{c} R_{x}\\ R_{y}\\ R_{z} \end{array}\right]=\left[\begin{array}{c} R_{x}^{E}+\frac{2(az+xy)}{a^{2}-x^{2}+y^{2}-z^{2}}R_{y}^{N}\\ R_{y}^{N}+\frac{2(xy-az)}{a^{2}+x^{2}-y^{2}-z^{2}}R_{x}^{E}\\ (-xR_{y}+yR_{x})/a \end{array}\right]$$

### 3.3. First-Order Acceleration Tracking

Since the ${\left[a,x,y,0\right]}^{T}$ component from the decomposition in Equation (10) directly determines the UAV’s acceleration, achieving a linear change in acceleration first requires the horizontal tilt to track a specific trajectory. This reference trajectory is constrained to have a yaw component that is zero.

The desired dynamics for $a_{E}$ are specified as a first-order response $\dot{a_{E}}=p\left(a_{Ed}-a_{E}\right)=-2p\left[{\left(ax\right)}_{d}-ax\right]$. However, the actual implemented dynamics in practice is Equation (17). Substituting into Equation (21) yields:

(22) $$\begin{array}{cc} \dot{a_{E}} & =R_{x}^{E}\left[-a^{2}+x^{2}-y^{2}+z^{2}+\frac{4(x^{2}y^{2}-a^{2}z^{2})}{a^{2}+x^{2}-y^{2}-z^{2}}\right]\\ \; & =-R_{x}^{E}\frac{\left(a^{2}-x^{2}-y^{2}\right)\left(a^{2}+x^{2}+y^{2}\right)}{a^{2}+x^{2}-y^{2}}\\ \; & =-\frac{2a^{2}-1}{2a^{2}+2x^{2}-1}R_{x}^{E}=-2p\left[{\left(ax\right)}_{d}-ax\right] \end{array}$$

Solving the equations yields the first-order control quantity:

(23) $$R_{x}^{E}=2p\left(\frac{2x^{2}}{2a^{2}-1}+1\right)\left[{\left(ax\right)}_{d}-ax\right]$$

Similarly, when $a_{D}$ follows the first-order trajectory $\dot{a_{N}}=p\left(a_{Nd}-a_{N}\right)=2p\left[{\left(ay\right)}_{d}-ay\right]$, we obtain:

(24) $$R_{y}^{N}=2p\left(\frac{2y^{2}}{2a^{2}-1}+1\right)\left[{\left(ay\right)}_{d}-ay\right]$$

### 3.4. Body-Frame Attitude Velocity

From the angular velocity ${\left[0,R_{x},R_{y},R_{z}\right]}^{T}$ of the horizontal tilt trajectory, we need to obtain the body-frame angular velocity ${\left[0,\omega_{x},\omega_{y},\omega_{z}\right]}^{T}$.

For the tilt-yaw decomposition attitude presented in Section 3.3, applying an angular velocity ${\left[0,R_{x},R_{y},R_{z}\right]}^{T}$ to the horizontal tilt over an infinitesimal time interval Δt yields a new vehicle attitude expressed as:

(25) $$\begin{array}{c} {\left[\begin{array}{cccc} a & x & y & 0 \end{array}\right]}^{T}⊗{\left[\begin{array}{cccc} c_{1} & \Delta x & \Delta y & \Delta z \end{array}\right]}^{T}⊗{\left[\begin{array}{cccc} b & 0 & 0 & z \end{array}\right]}^{T}\\ with\left[\begin{array}{c} c_{1}\\ \Delta_{x}\\ \Delta_{y}\\ \Delta_{z} \end{array}\right]=\left[\begin{array}{c} \sqrt{1-\Delta_{x}^{2}-\Delta_{y}^{2}-\Delta_{z}^{2}}\\ R_{x}\cdot \Delta t\\ R_{y}\cdot \Delta t\\ R_{z}\cdot \Delta t \end{array}\right] \end{array}$$

When the vehicle rotates with an angular velocity ${\left[0,\omega_{x},\omega_{y},\omega_{z}\right]}^{T}$, its attitude after an infinitesimal time step dt is represented as:

(26) $$\begin{array}{c} {\left[\begin{array}{cccc} w_{0} & x_{0} & y_{0} & z_{0} \end{array}\right]}^{T}⊗\left[\begin{array}{cccc} c_{2} & \delta_{x} & \delta_{y} & \delta_{z} \end{array}\right]\\ with\left[\begin{array}{c} c_{2}\\ \delta_{x}\\ \delta_{y}\\ \delta_{z} \end{array}\right]=\left[\begin{array}{c} \sqrt{1-\delta_{x}^{2}-\delta_{y}^{2}-\delta_{z}^{2}}\\ \omega_{x}\cdot \Delta t\\ \omega_{y}\cdot \Delta t\\ \omega_{z}\cdot \Delta t \end{array}\right] \end{array}$$

Because Equations (25) and (26) correspond to the same attitude, the two expressions are equivalent. Equating them and substituting the result into Equation (9), the solution is obtained as:

(27) $$\begin{array}{cc} bR_{x}+zR_{y} & =b\omega_{x}-z\omega_{y}\\ bR_{y}-zR_{x} & =b\omega_{y}+z\omega_{x}\\ R_{z} & =\omega_{z} \end{array}$$

Since $b^{2}+z^{2}=1$, it follows that:

(28) $$\left[\begin{array}{c} \omega_{x}\\ \omega_{y}\\ \omega_{z} \end{array}\right]=\left[\begin{array}{c} \left(b^{2}-z^{2}\right)R_{x}+2bzR_{y}\\ \left(b^{2}-z^{2}\right)R_{y}-2bzR_{x}\\ R_{z} \end{array}\right]$$

### 3.5. Angular Rate Limit

In practical deployment, maximum angular rates and attitude angles are always constrained in the controller design due to limitations in the UAV’s structural strength and actuators. For VTOL platforms, the roll and pitch axes are typically controlled by coupled actuators (e.g., the swashplate servos of a helicopter or the control allocation of a quadrotor). Therefore, we consider the maximum angular rate limit to be $\omega_{x}^{2}+\omega_{y}^{2}\leq W_{M}^{2}$, the maximum angular limit to be $x^{2}+y^{2}\leq A_{M}^{2}$ in Equation (13).

Considering Equations (28) and (21), $z=0,a^{2}+x^{2}+y^{2}=1$:

(29) $$\begin{array}{cc} \omega_{x}^{2}+\omega_{y}^{2} & ={\left(\left(b^{2}-z^{2}\right)R_{x}+2bzR_{y}\right)}^{2}+{\left(\left(b^{2}-z^{2}\right)R_{y}-2bzR_{x}\right)}^{2}\\ \; & =R_{x}^{2}+R_{y}^{2}\\ \; & ={\left(R_{x}^{E}+\frac{2(az+xy)}{a^{2}-x^{2}+y^{2}-z^{2}}R_{y}^{N}\right)}^{2}+{\left(R_{y}^{N}+\frac{2(xy-az)}{a^{2}+x^{2}-y^{2}-z^{2}}R_{x}^{E}\right)}^{2} \end{array}$$

Let

(30) $$e_{x}=2p\left[{\left(ax\right)}_{d}-ax\right],e_{y}=2p\left[{\left(ay\right)}_{d}-ay\right]$$

Considering Equations (23) and (24). Therefore, the angular-rate scaling factor *K* can be computed as follows:

(31) $$\begin{array}{cc} K & =\frac{\omega_{x}^{2}+\omega_{y}^{2}}{e_{x}^{2}+e_{y}^{2}}\\ \; & =\frac{{\left(1-2y^{2}\right)}^{2}e_{x}^{2}+{\left(1-2x^{2}\right)}^{2}e_{y}^{2}+{\left(2xy\right)}^{2}\left(e_{x}^{2}+e_{y}^{2}\right)+4xy\left(2-2x^{2}-2y^{2}\right)e_{x}e_{y}}{{\left(1-2x^{2}-2y^{2}\right)}^{2}\left(e_{x}^{2}+e_{y}^{2}\right)} \end{array}$$

By computing and processing *K*, it can be concluded that the maximum of *K* is independent of $W_{m}$ and depends only on $A_{m}$:

(32) $$K_{\max}=\frac{1}{{\left(1-2A_{m}^{2}\right)}^{2}}.$$

Detailed derivation is provided in Appendix A. Therefore, for UAV maneuvers where the attitude angle is constrained within $x^{2}+y^{2}\leq A_{M}^{2}$, it is sufficient to limit the rate of change of the acceleration coefficient:

(33) $${\left(e_{x}^{2}+e_{y}^{2}\right)}_{max}\leq {\left[W_{M}\left(1-2A_{M}^{2}\right)\right]}^{2}$$

This ensures that the angular velocity remains within its prescribed limits during attitude planning, while also achieving first-order tracking of the acceleration coefficient with bounded rate of change.

Certainly, under the same angular-rate limit, a tighter attitude constraint (closer to 90°) imposes a more restrictive bound on the actual angular velocity, thereby requiring a smaller allowed rate of change for the acceleration coefficients. However, as the allowed attitude range moves away from 90°, the corresponding scaling factor $K_{\max}$ decreases rapidly. For instance, with a limit of 60°, $K_{\max}=4$; for 45°, $K_{\max}=2$; and for 30°, $K_{\max}=1.333$. These values demonstrate considerable practicality for real-world UAV flight environments. Alternatively, the constraint can be applied in a piecewise manner during actual flight operations.

## 4. Attitude Planning

### 4.1. Algorithm Construction

Thus, we have derived a technique to convert desired acceleration into attitude error and angular rate commands. A key advantage of this method is that it decouples the problem from horizontal acceleration disturbances due to attitude changes. Additionally, it calculates an attitude trajectory that achieves first-order tracking of the target acceleration from current attitude.

The desired yaw angle $q_{zd}={\left[b_{d},0,0,z_{d}\right]}^{T}$ for the UAV is specified by either a navigation path or a remote command. Using the $q_{z}={\left[b,0,0,z\right]}^{T}$ to represent the current yaw attitude, the yaw error quaternion is computed as $q_{ze}=q_{zd}⊗q_{z}^{-1}$ and combined with the output $\omega_{zd}$ from Algorithm 1.

The desired total thrust *T* is determined by the outer-loop controller and is subsequently transmitted to the inner-loop controller to execute closed-loop tracking of the thrust command. For the purposes of outer-loop analysis and design, the closed-loop dynamics of the inner thrust control system are sufficiently characterized by a first or second-order approximation $f_{T}\left(T_{d},T\right)$. And the inner angular rate loop’s dynamics can be simplified to a system with negligible transients (approximated as instantaneous).
**Algorithm 1:** Attitude Planning Algorithm**Input:** 
Current attitude $q={\left[w_{0},x_{0},y_{0},z_{0}\right]}^{T}$; Desired value $\left[ax_{d},ay_{d}\right]$;**Output:** 
Target attitude $\left[a_{d},x_{d},y_{d}\right]$; Desired angular rate $\left[\omega_{xd},\omega_{yd},\omega_{zd}\right]$;1:Evaluate ${\left(e_{x}^{2}+e_{y}^{2}\right)}_{max}$ using $\left[W_{M},A_{M}\right]$ by Equation (33); Obtain the control limits under the current angular velocity and angle constraints;2:Evaluate [a,b,x,y,z] using q by Equation (9); Obtain the decomposition parameters of the current attitude;3:Evaluate $R_{x}^{E}$ using $\left[ax_{d},a,x\right]$ by Equation (23); Obtain the rotation amount generated by the desired eastward acceleration coefficient;4:Evaluate $R_{y}^{N}$ using $\left[ay_{d},a,y\right]$ by Equation (24); Obtain the rotation amount generated by the desired northward acceleration coefficient;5:Evaluate $e_{x}^{2}+e_{y}^{2}$ using $\left[ax_{d},ay_{d}\right]$ by Equation (30), if the limit is exceeded, $\left[R_{x}^{E},R_{y}^{N}\right]$ is scaled down proportionally; Obtain the rotation amount after applying the limits;6:Evaluate $\left[R_{x},R_{y},R_{z}\right]$ using $\left[R_{x}^{E},R_{x}^{E},a,x,y\right]$ by Equation (21); Obtain the rotation amount after first-order approximation compensation;7:Evaluate $\left[\omega_{xd},\omega_{yd},\omega_{zd}\right]$ using $\left[R_{x},R_{y},R_{z},b,z\right]$ by Equation (28); Convert to the rotation amount in the body coordinate system.

After these processing steps, substituting into Equation (12) and replacing $\left[ax,ay,ax_{d},ay_{d}\right]$ with $\left[q_{ax},q_{ay},q_{axd},q_{ayd}\right]$ to express it as independent variables, the outer-loop model of the system is simplified as:

(34) $$\left[\begin{array}{c} \dot{P}\\ \dot{u}\\ \dot{v}\\ \dot{w}\\ \dot{q_{ax}}\\ \dot{q_{ay}}\\ \dot{T} \end{array}\right]=\left[\begin{array}{c} V\\ T/m\cdot 2q_{ax}\\ -T/m\cdot 2q_{ay}\\ g-T/m\cdot \sqrt{1-4q_{ax}^{2}-q_{ay}^{2}}\\ p(q_{axd}-q_{ax})\\ p(q_{ayd}-q_{ay})\\ f_{T}\left(T_{d},T\right) \end{array}\right]$$

With $V={\left[u,v,w\right]}^{T}$.

### 4.2. Commonly Used Method

In UAV attitude control, both Euler angles and quaternions are widely adopted for attitude representation. When using Euler angles, the standard approach to generate attitude error and angular rate commands from desired acceleration is:

(35) $$\phi_{e}=\phi_{d}-\phi ,\theta_{e}=\theta_{d}-\theta ,\psi_{e}=\psi_{d}-\psi$$

By employing a front-right-down right-handed coordinate system and a yaw-pitch-roll rotation sequence, and substituting the corresponding attitude kinematics, the desired angular velocity is obtained as:

(36) $$\left[\begin{array}{c} \Delta_{x}\\ \Delta_{y}\\ \Delta_{z} \end{array}\right]=\left[\begin{array}{ccc} 1 & 0 & -\sin\theta \\ 0 & \cos\phi & \sin\phi \cos\theta \\ 0 & -\sin\phi & \cos\phi \cos\theta \end{array}\right]\left[\begin{array}{c} \phi_{d}-\phi \\ \theta_{d}-\theta \\ \psi_{d}-\psi \end{array}\right]$$

Under Euler angles, the commonly used outer-loop NMPC model is:

(37) $$\left[\begin{array}{c} \dot{P}\\ \dot{u}\\ \dot{v}\\ \dot{w}\\ \dot{\phi }\\ \dot{\theta }\\ \dot{\psi }\\ \dot{T} \end{array}\right]=\left[\begin{array}{c} V\\ T/m\cdot \cos\psi \sin\theta \cos\phi +\sin\psi \sin\phi \\ -T/m\cdot \sin\psi \sin\theta \cos\phi -\cos\psi \sin\phi \\ g-T/m\cdot \cos\theta \cos\phi \\ \omega_{x}\\ \omega_{y}\cos\phi +r\sin\phi \\ \omega_{y}\sin\phi /\cos\theta -r\cos\phi /\sin\theta \\ f_{T}\left(T_{d},T\right) \end{array}\right],\left[\begin{array}{c} \omega_{x}\\ \omega_{y}\\ \omega_{z} \end{array}\right]=\left[\begin{array}{c} p_{1}\Delta_{x}\\ p_{1}\Delta_{y}\\ p_{2}\Delta_{z} \end{array}\right]$$

When using quaternions, the standard approach to generate attitude error and angular rate commands from desired acceleration is:

(38) $$q_{e}=q_{d}⊗q^{-1}={\left[w_{e},x_{e},y_{e},z_{e}\right]}^{T}$$

If the scalar part of the error quaternion $w_{e}<0$, all components of $q_{e}$ are negated. This ensures that the error quaternion represents the shortest rotational path. The vector part of the attitude error quaternion is extracted to form the error vector that is used as the input to the controller ${\left[\Delta_{x},\Delta_{y},\Delta_{z}\right]}^{T}={\left[x_{e},y_{e},z_{e}\right]}^{T}$.

The difference between the desired angular velocity ${\left[\omega_{x},\omega_{y},\omega_{z}\right]}^{T}={\left[p_{1}\Delta_{x},p_{1}\Delta_{y},p_{2}\Delta_{z}\right]}^{T}$ and the current angular velocity is computed and fed into a P or PD controller. This is a commonly used method for attitude trajectory tracking.

Under quaternion, the commonly used outer-loop NMPC model is:

(39) $$\left[\begin{array}{c} \dot{P}\\ \dot{u}\\ \dot{v}\\ \dot{w}\\ \dot{q_{w}}\\ \dot{q_{x}}\\ \dot{q_{y}}\\ \dot{q_{z}}\\ \dot{T} \end{array}\right]=\left[\begin{array}{c} V\\ T/m\cdot 2(q_{w}q_{y}+q_{x}q_{z})\\ T/m\cdot 2(q_{y}q_{z}-q_{w}q_{x})\\ g-T/m\cdot (1-2q_{x}^{2}-2q_{y}^{2})\\ -q_{x}\omega_{x}-q_{y}\omega_{y}-q_{z}\omega_{z}\\ q_{w}\omega_{x}-q_{z}\omega_{y}+q_{y}\omega_{z}\\ q_{z}\omega_{x}+q_{w}\omega_{y}-q_{x}\omega_{z}\\ -q_{y}\omega_{x}+q_{x}\omega_{y}+q_{w}\omega_{z}\\ f_{T}\left(T_{d},T\right) \end{array}\right],\left[\begin{array}{c} \omega_{x}\\ \omega_{y}\\ \omega_{z} \end{array}\right]=\left[\begin{array}{cccc} 0 & p_{1} & 0 & 0\\ 0 & 0 & p_{1} & 0\\ 0 & 0 & 0 & p_{2} \end{array}\right]\left(q_{d}⊗q^{-1}\right)$$

Comparing the NMPC outer-loop models of the three methods, i.e., Equations (34), (37) and (39). Compared with the other two methods, the method proposed in this paper reduces both the number of parameters and the model complexity, which is beneficial for reducing the computational burden of NMPC optimization. Compared with the Euler-angle-based outer-loop model, it reduces one state variable and eliminates a large number of trigonometric function calculations. Compared with the quaternion-based outer-loop model, it reduces two state variables and significantly lowers the complexity of attitude estimation. Due to the unit norm constraint of quaternions, the complexity of attitude estimation is difficult to simplify.

## 5. Simulation and Results Analysis

Referring to the kinematic and dynamic models of the UAV, the dynamic response of the body angular velocity loop tracking is temporarily ignored for the validation of attitude trajectory planning. Taking the quaternion-based attitude representation as an example, this mathematical model defines ${\left[V,q\right]}^{T}$ as the system state variables and ${\left[\omega_{x},\omega_{y},\omega_{z},T\right]}^{T}$ as the system input variables. The attitude controller computes the planned value ${\left[\omega_{x},\omega_{y},\omega_{z}\right]}^{T}$ as the system input, while the UAV thrust *T* is controlled in a closed-loop manner by the outer-loop controller, which does not affect the simulation verification results.

According to the design objectives, we will validate the proposed method in this paper, along with the quaternion method and Euler angle method in Section 4.2. The dynamic response of the horizontal plane acceleration coefficient ${\left[aN,aE\right]}^{T}={\left[u/\left(T/m\right),v/\left(T/m\right)\right]}^{T}$, from the current state to the target setting, will be derived from Equation (6). Under the assumptions of no inverted flight and a given heading angle (where the heading angle representation follows the decomposition defined in Equation (13)), the attitude corresponds uniquely to the north-east acceleration coefficient. Since we intend the attitude trajectory to act as a first-order follower to the acceleration, we will compute the step response under various attitude conditions. The following simulations will be conducted at a control frequency of 1000 Hz, obtaining trajectory plots and time-domain evolution diagrams of the horizontal acceleration coefficient under attitude control using multiple methods, followed by analysis.

**Figure 2:**
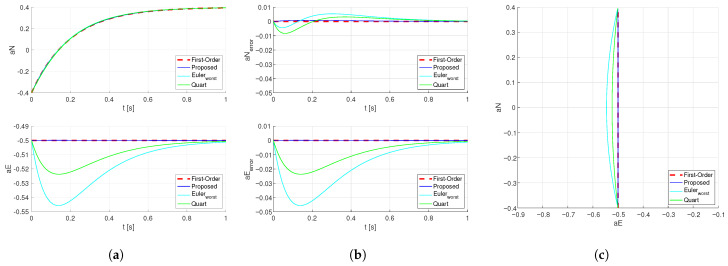
Step Response of the Horizontal Acceleration Coefficient. (**a**) Dynamic response of the horizontal acceleration coefficients subject to different attitude planning schemes. (**b**) Dynamic response of the deviation in horizontal acceleration coefficients from the first-order reference under different attitude planning schemes. (**c**) Trajectories of the horizontal acceleration coefficients under different attitude planning methods.

With identical control gain P and without considering the maximum angular rate limit, the horizontal acceleration coefficient is calculated from the current state ${\left[aN,aE\right]}^{T}={\left[-0.4,-0.5\right]}^{T}$ to the target ${\left[aN,aE\right]}^{T}={\left[0.4,-0.5\right]}^{T}$. The results for the quaternion-based method, the Euler-angle method, and the method proposed in this paper are as Figure 2.

**Figure 3:**
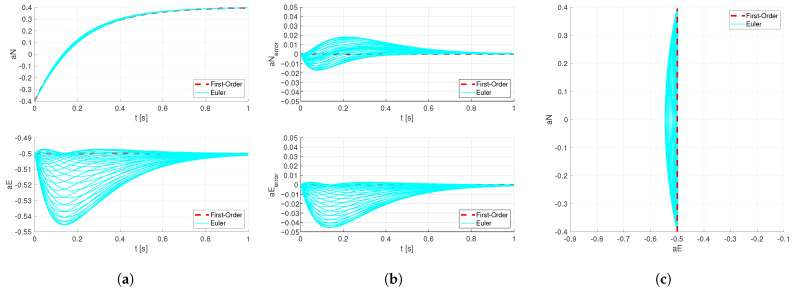
Step response of the horizontal acceleration coefficient for the Euler-angle method under multiple heading angles. (**a**) Dynamic response of the horizontal acceleration coefficients. (**b**) Dynamic response of the deviation in horizontal acceleration coefficients from the first-order reference. (**c**) Trajectories of the horizontal acceleration coefficients.

**Figure 4:**
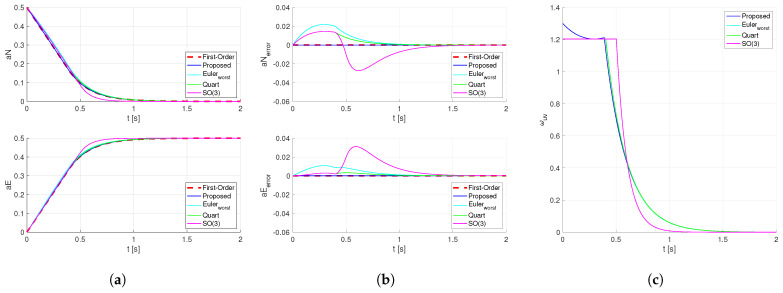
Step Response of the Horizontal Acceleration Coefficient with angular rate limit. (**a**) Dynamic response of the horizontal acceleration coefficients subject to different attitude planning schemes. (**b**) Dynamic response of the deviation in horizontal acceleration coefficients from the first-order reference under different attitude planning schemes. (**c**) The combined magnitude of the roll and pitch angular velocity components.

For any heading angle, both the quaternion method and the proposed method compute identical horizontal acceleration coefficient trajectories. Due to the use of the ‘Z-Y-X’ rotation sequence in the Euler-angle method, its dynamic response varies under different heading angles. In the results plotted in Figure 2, the worst-case scenarios are selected for illustration. The responses of the Euler-angle method, calculated at heading angle intervals of π/36, are shown below as Figure 3:

With identical control gain P and considering the maximum angular rate limit $\omega_{max}=pi/2$, we compute the transition from the current North-East acceleration coefficient ${\left[aN,aE\right]}^{T}={\left[0.5,0\right]}^{T}$ to the target ${\left[aN,aE\right]}^{T}={\left[0,0.5\right]}^{T}$ for the quaternion method, the Euler angle method, and the method proposed in this paper. Additionally include an SO(3)-based attitude controller using $e=\log q_{e}$ as attitude error under the same gains for comparison. The results are shown below as Figure 4:

Due to the use of the ‘Z-Y-X’ rotation sequence in the Euler-angle method, its dynamic response varies under different heading angles. In the optimal case, its trajectory approximates that of the proposed method. The worst-case scenarios are selected for illustration in the Figure 4.

Based on the experimental results presented above, if we regard the acceleration coefficient as a spherical surface, the attitude control trajectory planned by the quaternion method and SO(3) method corresponds to the shortest path on the sphere. The results shown in Figure 4 indicate that when using the SO(3) method, the target attitude is achieved more quickly in the time domain. In contrast, the attitude control trajectory planned by the method proposed in this paper is a straight line projected onto the horizontal plane, which also satisfies first-order tracking of the target horizontal acceleration in the time domain. Minor errors arise from the nonlinear mapping introduced during discretization in the control process.

**Figure 5:**
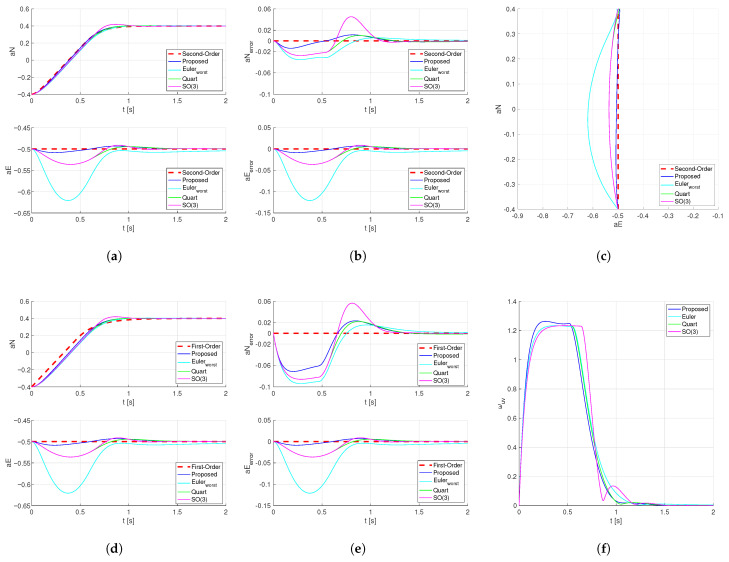
Step response of the horizontal acceleration coefficient under different attitude planning schemes for the complete inner-loop model. (**a**) orizontal acceleration coefficient and the second-order reference. (**b**) Deviation between the horizontal acceleration coefficient and the second-order reference. (**c**) Trajectory of the horizontal acceleration coefficient. (**d**) Horizontal acceleration coefficient and the first-order reference. (**e**) Deviation between the horizontal acceleration coefficient and the first-order reference. (**f**) Sum of the roll angular velocity component and the pitch angular velocity component.

However, in practical applications, the inner loop is non-ideal, and the angular velocity cannot achieve the desired target value in real time. Considering the complete rigid-body rotation (including gyroscopic torque, aerodynamic damping, and control torque), the angular velocity controller adopts a common PI controller with feedforward. Under the condition of amplitude limiting, the attitude is controlled from ${\left[aN,aE\right]}^{T}={\left[-0.4,-0.5\right]}^{T}$ to the target ${\left[aN,aE\right]}^{T}={\left[0.4,-0.5\right]}^{T}$ (same as in Simulation 1). The actual values of the acceleration coefficient are compared with the first-order reference and the second-order reference, respectively. The results are shown below as Figure 5:

In fact, the complete attitude mathematical model with the controller is third-order nonlinear. However, for outer-loop control estimation, the full third-order estimation is rarely used. Therefore, common first-order and second-order references are selected for comparison. From the acceleration coefficient aE in Figure 5, it can be seen that the control method proposed in this paper achieves the best decoupling performance for the horizontal acceleration coefficient. From the acceleration coefficient aN in Figure 5, it can be seen that the proposed control method consistently exhibits the smallest deviation when compared with either the first-order or second-order reference, and a significant improvement is achieved when using the second-order reference.

## 6. Conclusions

Comprehensive comparison shows that, under the limit of attitude and angle-rate constraint, the angular velocity profile generated by the attitude trajectory control method proposed in this paper achieves excellent first-order tracking of the target horizontal acceleration coefficient. This approach simplifies the outer-loop model and provides a high-quality estimation of the acceleration (attitude) dynamic response.

The next step will involve incorporating angular rate limits and first-order angular rate dynamics to construct a refined inner-loop dynamic response model, thereby improving the performance of outer-loop trajectory tracking control.

## Data Availability

The simulation algorithm developed in this study is openly available on GitHub at https://github.com/ericxd1997/AttitudeTrajectory (accessed on 20 April 2026). No additional data were used in this research.

## Abbreviations

The following abbreviations are used in this manuscript:

UAV	Unmanned Aerial Vehicle
VTOL	Vertical Take-Off and Landing

## Appendix A. The Maximum of Angular-Rate Scaling Factor

Given

$$F=\frac{{\left(1-2y^{2}\right)}^{2}e_{x}^{2}+{\left(1-2x^{2}\right)}^{2}e_{y}^{2}+{\left(2xy\right)}^{2}\left(e_{x}^{2}+e_{y}^{2}\right)+4xy\left(2-2x^{2}-2y^{2}\right)e_{x}e_{y}}{{\left(1-2x^{2}-2y^{2}\right)}^{2}\left(e_{x}^{2}+e_{y}^{2}\right)}.$$

$$x=A_{m}\sin t_{1},\;y=A_{m}\cos t_{1},\;e_{x}=W_{m}\sin t_{2},\;e_{y}=W_{m}\cos t_{2}$$

where

$$-\pi <t_{1}\leq \pi ,\;-\pi <t_{2}\leq \pi ,\;0<A_{m}<\frac{\pi }{2},\;0<W_{m}<\frac{\pi }{2}.$$

We aim to show that the maximum of *F* is independent of $W_{m}$ and to find its relation with $A_{m}$.

From

$$x^{2}+y^{2}=A_{m}^{2}\left(\sin^{2}t_{1}+\cos^{2}t_{1}\right)=A_{m}^{2}\equiv A,$$

$$e_{x}^{2}+e_{y}^{2}=W_{m}^{2}\left(\sin^{2}t_{2}+\cos^{2}t_{2}\right)=W_{m}^{2}\equiv W.$$

Let $u=\sin t_{2},v=\cos t_{2},u^{2}+v^{2}=1$, so

$$e_{x}=\sqrt{W}\;u,\;e_{y}=\sqrt{W}\;v.$$

The denominator becomes

$${\left(1-2x^{2}-2y^{2}\right)}^{2}\left(e_{x}^{2}+e_{y}^{2}\right)={\left(1-2A\right)}^{2}W.$$

Substituting into the numerator, thus the numerator is

$$W\left[{\left(1-2y^{2}\right)}^{2}u^{2}+{\left(1-2x^{2}\right)}^{2}v^{2}+4x^{2}y^{2}+4xy\left(2-2A\right)uv\right].$$

Eliminating *W*

$$F=\frac{{\left(1-2y^{2}\right)}^{2}u^{2}+{\left(1-2x^{2}\right)}^{2}v^{2}+4x^{2}y^{2}+4xy\left(2-2A\right)uv}{{\left(1-2A\right)}^{2}}.$$

This expression is independent of $W_{m}$. Hence, the maximum of *F* is independent of $W_{m}$, establishing the first part of the claim.

Maximizing over $t_{1},t_{2}$ for fixed $A_{m}$

Let $C=\cos2t_{1}$, $S=\sin2t_{1}$, with $C^{2}+S^{2}=1$. From $x=\sqrt{A}\sin t_{1}$, $y=\sqrt{A}\cos t_{1}$ we have:

$$1-2y^{2}=1-A\left(1+C\right)\equiv a,\;1-2x^{2}=1-A\left(1-C\right)\equiv b,$$

$$xy=\frac{A}{2}S,\;x^{2}y^{2}=\frac{A^{2}}{4}S^{2}.$$

Thus

$$a+b=2-2A,\;b-a=2AC,\;a^{2}+b^{2}=2{\left(1-A\right)}^{2}+2A^{2}C^{2}.$$

Let $\phi =t_{2}$, so u=sinϕ, v=cosϕ. Define *Q* as the numerator of *F* excluding the factor ${\left(1-2A\right)}^{2}$:

$$Q=a^{2}u^{2}+b^{2}v^{2}+A^{2}S^{2}+4A\left(1-A\right)S\;uv.$$

Using $u^{2}=\frac{1-\cos2\phi }{2}$, $v^{2}=\frac{1+\cos2\phi }{2}$, $uv=\frac{\sin2\phi }{2}$:

$$Q=\frac{a^{2}+b^{2}}{2}+\frac{b^{2}-a^{2}}{2}\cos2\phi +A^{2}S^{2}+2A\left(1-A\right)S\sin2\phi .$$

Substituting $b^{2}-a^{2}=4AC\left(1-A\right)$ and $a^{2}+b^{2}=2{\left(1-A\right)}^{2}+2A^{2}\left(1-S^{2}\right)$:

$$\begin{array}{cc} \frac{a^{2}+b^{2}}{2}+A^{2}S^{2} & ={\left(1-A\right)}^{2}+A^{2}\left(1-S^{2}\right)+A^{2}S^{2}\\ \; & =1-2A+2A^{2}. \end{array}$$

Thus

$$Q=1-2A+2A^{2}+2AC\left(1-A\right)\cos2\phi +2A\left(1-A\right)S\sin2\phi .$$

Maximizing over ϕ. Let $P\left(\phi \right)=2A\left(1-A\right)\left[C\cos2\phi +S\sin2\phi \right]$. Its maximum is

$$\max_{\phi }P\left(\phi \right)=2A\left(1-A\right)\sqrt{C^{2}+S^{2}}=2A\left(1-A\right).$$

Hence

$$\max_{\phi }Q=1-2A+2A^{2}+2A\left(1-A\right)=1.$$

This value is independent of $t_{1}$ (i.e., independent of C,S). Therefore, for any $t_{1}$, we can choose ϕ to achieve Q=1.

Maximum of *F*

$$F_{\max}=\frac{\max Q}{{\left(1-2A\right)}^{2}}=\frac{1}{{\left(1-2A_{m}^{2}\right)}^{2}}.$$

The maximum is independent of $W_{m}$ and depends only on $A_{m}$.
